# Conceptualizing and Measuring Systemic Racism

**DOI:** 10.1146/annurev-publhealth-060222-032022

**Published:** 2025-01-14

**Authors:** Tyson H. Brown, Hedwig E. Lee, Margaret T. Hicken, Eduardo Bonilla-Silva, Patricia Homan

**Affiliations:** 1 Department of Sociology, Duke University, Durham, North Carolina, USA; 2 Institute for Social Research, University of Michigan, Ann Arbor, Michigan, USA; 3 Department of Sociology, Florida State University, Tallahassee, Florida, USA

**Keywords:** health disparities, measurement, methods, theory, structural racism, systemic racism

## Abstract

This article provides a guide for rigorous, theory-driven measurement approaches, proposing best practices for the scientific study of systemic racism in health research. We argue that the analytical crux of measuring systemic racism—a complex, interconnected, and dynamic system—lies in operationalizing the collective logics, properties, and mechanisms that undergird racial inequities. Misalignment between measurement tools and these fundamental features undermines research validity, as incongruent measures distort findings and obscure systemic racism’s true impacts. To address this, we draw on interdisciplinary theories and evidence to dissect key features of systemic racism, emphasizing their implications for measurement. We further recommend incorporating temporal processes in health research by leveraging core principles of the life course perspective, which elucidate the intricate interplay of individual, historical, and societal trajectories. Our recommendations underscore the necessity of adopting robust, evidence-based methods to advance the understanding of how systemic racism operates and shapes public health.

## 1. INTRODUCTION

Systemic racism is a matter of life and death. Addressing systemic racism is essential for achieving health equity and improving population health, highlighting the critical need for an accurate and robust scientific foundation on how systemic racism operates and harms health (20–22). Recent years have witnessed a surge in public health research on systemic racism, fueled by heightened public awareness, expanded funding opportunities, and the proliferation of special journal issues dedicated to this pressing topic (71, 79). However, the rapid pace of this research has often prioritized volume over rigor, leading to studies that address superficial and mimetic research questions and to the operationalization of systemic racism in ways that are not theoretically or empirically sound. Common pitfalls include a failure to account for its dynamic, multifaceted, and interconnected nature (68, 79, 86). These limitations in conceptualization and measurement have contributed to fragmented and inconsistent findings, ultimately impeding progress toward a rigorous and comprehensive understanding of how systemic racism shapes public health outcomes (see the sidebar titled What Is Systemic Racism?).

This review article serves as a synthesis and field guide for quantitative public health research on racism, illuminating limitations of and gaps in the literature while proposing theory-driven best practices for conceptualizing and measuring systemic racism. Complementing and building on prior agenda-setting studies on methods for studying racism (2, 6, 8, 20, 22, 26, 33, 51, 57, 68, 69, 74, 78, 85, 127, 128), the goal of this article is to stimulate rigorous empirical research that examines deep and salient questions about the impact of systemic racism on public health. First, we draw on foundational race theories from the humanities and social sciences to develop a rich conceptualization of systemic racism, with a focus on its key properties; we propose novel approaches to measuring and modeling systemic racism in ways that align with these features (Table 1 provides a summary). Second, given that systemic racism is a complex and dynamic phenomenon that unfolds over time, we recommend ways to incorporate temporal processes in health research on systemic racism by applying core principles of the life course perspective. The life course perspective highlights the intricate interplay of biographical, historical, and societal trajectories, emphasizing various dimensions of temporality such as age, period, cohort, and intergenerational factors. Whereas prior research has often ignored critical issues of temporality—which has hindered accurate operationalization of systemic racism—this article provides concrete examples of how to integrate life course approaches for conceptualizing, measuring, and modeling systemic racism’s effects on health (see Table 2). Third, we illuminate several additional key issues related to the measurement of systemic racism. We conclude with a summary of best practices and recommendations for how future research can be more conceptually and analytically rigorous in order to build a strong evidence base on the health effects of systemic racism. The literature we draw on and the examples we cite come primarily from the US context, but the general features of systemic racism, the application of life course principles, and the best practices we identify are largely applicable across different contexts.

## 2. ALIGNING SYSTEMIC RACISM MEASUREMENT WITH THEORY

Although the measurement of systemic racism in public health is a relatively recent endeavor, humanists and social scientists have been conceptualizing and analyzing systemic racism and its consequences for more than a century (13, 27, 40, 54, 95, 121, 123). This extensive body of work provides a robust theoretical foundation for identifying the key features of systemic racism necessary for its empirical operationalization. Effective linkage between theory and measurement requires more than a definition of systemic racism—it necessitates a thorough dissection. Such dissection yields critical insights into not only the persistence and pervasiveness of systemic racism but also the mechanisms through which it influences health outcomes. Drawing on theoretical and empirical evidence, we outline the key features of systemic racism—including relational power dynamics, historical roots and collective amnesia, racialized logics and ideologies, institutional manifestations, its ubiquitous and multifaceted nature, and its interconnected and adaptive networks—and offer specific recommendations for integrating these features into measurement and modeling practices. Table 1 summarizes these features and presents best practices for their operationalization as well as guidelines for addressing salient research questions.

### 2.1. Relational Hierarchies and Power Dynamics

Theory underscores systemic racism as a fundamental cause of racialized health inequities (102). A central tenet of race theories is that racialized social systems are characterized by relational hierarchies and power dynamics (10, 13, 40, 48, 98, 121). Racial hierarchies undergird a society’s racial structure, driven by racial projects that generate material advantages for the dominant race and disadvantages for subaltern races (16, 100). This process involves the formation of socially constructed racial categories themselves, which vary across sociohistorical contexts (100). While race is a biological fiction, the social construction of racial groups has very real consequences for nearly all facets of society and aspects of individuals’ lives, resulting in the unequal allocation of power, risks, resources, rights, and opportunities, such as access to education, employment, housing, health care, legal protections, and political power (111, 129). Indeed, Seamster & Ray (114) observe, “Whites’ well-being is the manifestation of a hierarchical system. Whites are doing better because the structural relations of race benefit, reward, and empower them” (p. 333). Racialized actors (individuals, organizations, institutions, and the state) wield influence in society in many ways, including violence, economic means, cultural hegemony, political and legal structures (e.g., social and health policies), and other mechanisms (5, 6, 17, 38, 51, 94, 95). Notably, despite evolutions in racialized hierarchies and power dynamics in the United States over time, White supremacy has been a constant, leading to persistent racial inequalities in life chances, including, but not limited to, health (102).

#### 2.1.1. Implications for measurement.

Insights from race theories suggest that measuring racialized hierarchies and power dynamics requires a multipronged approach that combines various data sources and methods to capture the complexity and multidimensionality of race relations. Power structure analysis is particularly relevant because it focuses on how power is distributed, exercised, and perceived within societies (38). For example, by leveraging organizational data, surveys, network analysis, content analysis, and comparative designs, researchers can systematically quantify and analyze the roles that specific racialized actors play in contributing to racial stratification (16, 38, 88, 116). Other promising approaches include, but are not limited to, examining the impacts of policies (e.g., laws related to affirmative action and welfare) to reveal how they contribute to racial stratification; utilizing experiments, surveys, and digital trace data to investigate racial ideologies (14, 93) and biases (24, 66); and using administrative data to study how hegemonic racial subordination and superordination manifest in racial inequalities in power and life chances (e.g., political representation, disenfranchisement, poverty, incarceration, and segregation) (21, 90).

### 2.2. Historical Roots and Amnesia

In sharp contrast to dominant narratives of racial progress, which rely on teleological assumptions grounded in color-blind racism and Enlightenment thought (114), race theories emphasize how the enduring legacies of historical racism continue to shape social, economic, and political structures, resulting in systemic racial inequalities that persist across time (1, 9, 15, 100, 112). As scholars have demonstrated, these persistent inequities are rooted in the institutionalization of racial hierarchies, a phenomenon perpetuated through both explicit legal frameworks and more insidious, covert social practices (4, 10, 41, 48). Contemporary racial inequities in health, and their social, economic, and political drivers, are linked to the past in two ways. First, historical racial structures and processes set trajectories in motion to the present. For example, research suggests a link between place-based histories of enslavement and contemporary patterns in heart disease mortality among Black people in the United States, a relationship theorized to stem, in part, from intervening histories of lynching and racial inequalities in socioeconomic resources (77). Second, the same fallacious ideology—that some racialized groups are disposable while others are worthy of investment—has driven historical institutions and continues to shape present-day ones (95, 101). Notably, public health research often minimizes or ignores the influence of history on contemporary racial health inequities.

#### 2.2.1. Implications for measurement.

Interest is growing in explicitly measuring and modeling how contemporary health outcomes are shaped by various forms of historical racism, such as chattel slavery, lynchings, Jim Crow laws, sundown towns, redlining, and other types of racial oppression (3, 7, 34, 47, 55, 77, 80, 107, 109, 110). One of the main challenges to studying the relationship between historical context and contemporary health patterns is that individuals experiencing the measured health outcomes often did not live during that historical period, which underscores the importance of having conceptual and analytical clarity regarding exposures and potential intervening mechanisms. This challenge is particularly evident in the growing body of literature leveraging digitized Home Owners’ Loan Corporation (HOLC) maps to investigate the associations between residence in formerly redlined areas and contemporary health outcomes (84, 99). Indeed, researchers must ensure that these studies do not flatten historical processes and should account for the fact that there is sociospatial diversity in how cities have developed over the decades between the creation of HOLC maps and the present. For example, Graetz & Esposito (65) use sequential mediation models to test the extent to which the relationship between twentieth-century redlining and contemporary life expectancy is mediated by intervening racialized processes such as urban renewal, school segregation, and discriminatory race-based property valuation (65). By using methods that align with theory, Graetz & Esposito’s study made several important contributions, including (*a*) examining how racism is a historically contingent structural determinant of health, (*b*) situating redlining practices as one among numerous forms of historical racism in the political economy of racial stratification, and (*c*) estimating how twentieth-century redlining practices triggered a sequence of structurally racist practices that continue to affect contemporary racial phenomena. Moving forward, scholars should utilize theoretically informed data, measures, and methods (e.g., longitudinal, multilevel, and counterfactual models; sequential mediational analyses; Bayesian models; directed acyclic graphs) to comprehend the enduring health effects of a variety of historical racist contexts.

### 2.3. Racialized Schemas, Logics, and Ideologies

The racialized social system is the actualization of fallacious schemas, logics, and ideologies that some racial groups are superior to others (95). Racial cognitive frameworks are woven into the fabric of US culture through shared beliefs, values, norms, practices, symbols, and material objects that shape how people perceive, understand, and interact with the racial landscape (16, 26, 70, 96, 97, 105). Colorblind racism theory underscores how systemic racism in the post–Civil Rights Era is upheld and rationalized through several racial cognitive frames, including (*a*) abstract liberalism (e.g., using ideas associated with political and economic liberalism such as equal opportunity and individual choice to explain and justify racial matters in a way that ignores structural inequalities), (*b*) naturalization (normalizing racial phenomena through beliefs that they are natural and inevitable occurrences, rather than being socially constructed products of racism), (*c*) victim blaming (justifying racial inequality by using false racial tropes based on presumed fixed cultural deficiencies of non-White racial groups), and (*d*) minimization of racism (downplaying the significance of ongoing racial discrimination and inequality, often suggesting that racism is no longer a major factor affecting the lives of minorities) (15). Similarly, the theory of racial ignorance, rooted in critical race theory, argues that racial ignorance is not merely a lack of knowledge but a socially constructed and actively maintained state that serves to uphold racial hierarchies. It emphasizes how institutions and individuals purposefully ignore or misrepresent racial realities to protect systems of White supremacy (87, 97). While racialized schemas, logics, and ideologies are particularly challenging to recognize, explicate, and quantify because they are part of the collective social subconscious, it is essential to empirically study their role in shaping racial health inequalities.

#### 2.3.1. Implications for measurement.

The schemas, logics, and ideologies undergirding systemic racism necessitate careful consideration of appropriate measures, models, and interpretations of results (130). Since many features of systemic racism may appear neutral or rational on the surface, relying solely on measures of overt racial discrimination and violence is insufficient for fully capturing the complex links between systemic racism and health. While those remain important aspects of racism, there are many other measurable manifestations of fallacious racialized cognitive frameworks underlying White supremacy (20, 26, 69, 93). These phenomena can be measured in numerous ways, including, but not limited to, experimental studies on implicit racialized associations and biases (66); survey tools assessing color-blind ideologies, racial ignorance, antipathy, resentment, and apathy (52, 67, 118); leveraging of digital trace data on racism from social media and search engines (24); analysis of symbols of racial violence, such as localities, landmarks, and organizations named after notorious racists (93); studies of voting patterns tied to support for racist officials and policies (82, 92); and power structure analysis to uncover the roles of racialized actors in perpetuating racial stratification (38).

### 2.4. Institutional Manifestations

Racism is etched into the social, economic, and political institutions that structure society (74, 103, 116, 121). In fact, scholars argue that all US institutions are imbued with racism in one way or another (13, 35, 108, 121). Because racism is baked into the structure and functions of institutions, it does not require individual awareness or intent to persist (15, 16) and to harm the health of oppressed groups. These racialized structures perpetuate inequalities across sectors of society and over time through seemingly neutral policies, norms, and practices, often under the guise of fairness or meritocracy (95, 96, 106). As a result, racism remains deeply entrenched in everyday social life, shaping the inequitable distribution of opportunities and resources across the color line.

#### 2.4.1. Implications for measurement.

In empirically examining the health consequences of systemic racism, researchers most commonly employ metrics of racial residential segregation (considered by many scholars to be a lynchpin of the racialized social system), followed by indicators of racial inequities in institutional domains (e.g., economic, educational, criminal-legal, political, and health care systems) to indirectly gauge discrimination (68, 71, 78). While such proxy measures provide useful insights, these observed institutional inequities are, themselves, midstream consequences of institutional racism operating further upstream. One way to assess upstream forms of institutional racism more directly is to analyze discriminatory policies and practices, such as affirmative action bans, the prohibition on teaching critical race theory (106), redlining (91), wage theft (49), predatory lending (120), organized abandonment (63, 110), and xenophobic immigration policies (113). Directly examining policies and practices is especially well-suited for capturing how racism is “etched into the rules, norms and logics of institutions” (116, p. 404). Notably, studies that operationalize institutional forms of racism often focus on a single institutional domain, policy, or practice. While such targeted approaches may offer valuable insight into racism within an individual institution, they often come at the cost of understanding how racism operates across the broader, interconnected web of various institutions. Future theoretically informed, empirically based analyses should assess the relative trade-offs of these alternative approaches.

### 2.5. Ubiquitous and Multifaceted Nature

Systemic racism is defined by its ubiquity and entrenched presence across virtually every aspect of society (35, 114). Racial oppression is not a series of isolated incidents; rather, it operates as a pervasive system that shapes the structures and functions of social institutions (13, 85, 108). A wealth of research has demonstrated how systemic racism is embedded across a wide array of societal domains, including the political system (68, 75), environmental policies (104), the criminal-legal system (85), housing markets (76, 120), educational systems (62), technology sectors (11), the economy (32), health care institutions (47), and immigration law (113), among others. This omnipresence of racism across societal domains underscores its multifaceted nature, as it influences and reinforces racial inequalities in each of these areas. Despite the evolving expressions of racism, it remains a constant force shaping social realities and perpetuating inequities (58, 114). Understanding its multifaceted impact across these domains is essential for addressing its pervasive influence.

#### 2.5.1. Implications for measurement.

The pervasiveness of systemic racism implies that measures and models of the phenomenon should account for its multifaceted and relative nature. Scholars have recently argued that multifaceted measures that capture multiple institutions simultaneously (for example, latent scales of racism across several domains) can be useful for quantifying the health effects of systemic racism (2, 19, 21, 39, 71). For example, recent work has shown that a latent variable measure of systemic racism combining indicators of racism in seven different institutions had better model fit and explained substantially more variation in population health than did either observed individual indicators or a summative index of observed indicators (that assumed equal weighting of indicators and ignored correlations among them) (23). In addition, machine learning approaches likely have considerable utility for enhancing the study of systemic racism by analyzing vast amounts of data to uncover patterns of racism in various societal domains. Thus, latent variables and machine learning methods are useful approaches to measuring and modeling systemic racism, reflecting its multifaceted nature (2, 20, 21).

Moreover, given the pervasive nature of systemic racism, it is crucial to recognize that designating an environment as “low racism” based on specific measures does not necessarily imply that the environment is truly equitable. Instead, such a designation indicates that the observed manifestations of racism, as captured by a particular metric at a specific time and place, are comparatively lesser than those in other settings. Systemic racism is inherently relational and context dependent (30), necessitating careful consideration of what is reflected by measures of systemic racism. For instance, a setting that appears low in discriminatory economic practices may still exhibit relatively high levels of other forms of racism, such as legacies of racial violence or the pervasive influence of color-blind ideologies. Consequently, addressing the pervasive nature of systemic racism requires the development and application of broad, multifaceted metrics that capture racism across diverse domains. Moreover, it is essential to explicitly acknowledge the specific dimensions of racism that are captured—and those that are omitted—by any given measurement approach.

### 2.6. Interconnected and Adaptive Network

Systemic racism involves an interconnected and adaptive network of racist ideologies and institutions. Interconnectedness is a fundamental feature of systemic racism, reflecting how various cultural and institutional phenomena are mutually reinforcing in perpetuating racial inequalities (21, 69, 85, 95). When conceptualizing and operationalizing systemic racism, we need to understand that it is not isolated within one domain; rather, it operates and diffuses across multiple aspects of society, creating a complex and enduring web of advantages for White populations and disadvantages for racialized minorities (13, 85, 108). For instance, consider the interplay between racialized housing, education, and employment processes. Discriminatory housing policies and practices such as racialized rent exploitation, redlining, steering, blockbusting, predatory lending, and undervaluation have confined certain racial groups, particularly Black Americans, to under-resourced neighborhoods (31, 116, 120). These neighborhoods are often associated with lower rates of homeownership, lower home appraisals, and lower-quality schools due to property tax–based funding systems, which limit educational opportunities and outcomes for residents (46, 76). Poor educational attainment then restricts job opportunities, leading to lower income and wealth levels and reduced economic mobility (37). These economic constraints reinforce the initial housing disparities, creating a cyclical pattern of disadvantage (32, 46). Moreover, systemic racism is embedded within and perpetuated by cultural representations and societal norms. Media portrayals, for instance, often reinforce racialized narratives that influence public perceptions and attitudes toward different racial groups. These perceptions can shape policy preferences and legislative outcomes, further entrenching institutionalized racial inequalities.

Systemic racism is also adaptive, meaning that racial control can operate even when particular institutions or practices are no longer able to serve as modes of racial domination due to changes in laws or social norms across geographies and/or time (15, 102). Gee & Hicken (58) use the analogy of a “bucky ball” (Buckminsterfullerene) to describe this adaptive “system of interconnected institutions that operates with a set of racialized rules that maintain White supremacy. These connections and rules allow racism to reinvent itself into new forms and persist, despite civil rights interventions directed at specific institutions” (p. 293). The dynamism of systemic racism has been articulated by many critical race theorists. For example, Alexander (4), Flowe (50), Hinton (72), Muhammad (98), and Wacquant (122) have compellingly described how racial residential segregation and racial disproportionality in mass policing and incarceration can be linked to practices of Jim Crow and chattel slavery, working in tandem, symbiotically, or in lieu of one another as modes of racial control. In this way, systemic racism evolves rather than being eradicated.

#### 2.6.1. Implications for measurement.

Research on systemic racism must utilize measurement and modeling approaches that capture its interconnected and adaptive nature. Studies should examine dynamic temporal and spatial relationships and processes among the ideologies, policies, practices, and institutions implicated in the reproduction, reinforcement, and evolution of systemic racism. Agent-based network analysis and system dynamics modeling are promising approaches for studying systemic racism due to their utility in estimating and visualizing complex relationships and processes, such as diffusion, feedback loops, spillovers, and lagged effects (22, 85). These methods offer unique insights into how systemic racism evolves and persists across different contexts. In addition, machine learning techniques, with their ability to analyze large datasets and uncover intricate patterns, are particularly well-suited for investigating the interconnected and adaptive nature of systemic racism (22). Each of these approaches can reveal hidden relationships and dynamic shifts over time, providing a deeper understanding of how racism operates at macro, meso, and micro levels.

## 3. LIFE COURSE APPROACHES TO CONCEPTUALIZING AND MEASURING SYSTEMIC RACISM

Public health research on systemic racism has often overlooked critical issues related to temporality, limiting our ability to fully understand the dynamic processes through which systemic racism shapes health over time (20, 56, 59, 60, 79). Incorporating insights from the life course perspective offers an opportunity to address this gap and advance a more nuanced and comprehensive understanding of these processes. The life course perspective emphasizes the interplay of individual, historical, and societal trajectories, focusing on key dimensions of temporality such as age, period, cohort, and intergenerational dynamics (43, 45, 61). In the sections that follow, we outline five core principles of the life course—lifelong human development, timing in lives, sociohistorical locations, linked lives, and human agency—and discuss how applying these principles can strengthen the measurement and modeling of systemic racism’s health impacts. Table 2 provides a summary of these principles and offers guidance on how they can inform research examining the links between systemic racism and health.

### 3.1. Lifelong Human Development

The life course principle of lifelong human development underscores that (*a*) individual growth, change, and well-being are dynamic processes that extend from birth to death; (*b*) experiences and outcomes at one stage of life can influence subsequent stages; and (*c*) developmental processes are influenced by the complex interplay of individual and contextual factors (44, 45). This principle illustrates the importance of examining how systemic racism impacts health by shaping key life transitions (e.g., starting a job, buying a house, or becoming incarcerated), trajectories (e.g., long-term patterns of stability and change in various life domains), and cumulative disadvantage processes (over time, individuals accumulate experiences and exposures that can lead to advantages or disadvantages). For example, future research should empirically test the extent to which exposure to residential segregation limits Black children’s access to quality education, leading to a cascade of disadvantages such as reduced job prospects and opportunities for building wealth and increasing risks of incarceration and other stressors, all of which undermine health. This example illustrates how longitudinal approaches are critical for studying how exposure to systemic racism over the life course influences social, economic, and health trajectories.

### 3.2. Timing in Lives

While the life course principle of timing in lives emphasizes that the health effects of social conditions are contingent on temporal factors (e.g., age, sequencing, duration, and latency periods), we know very little about whether/how temporal factors moderate the effects of systemic racism on health (42, 60). Prior studies have revealed evidence of sensitive periods, that is, specific ages and developmental stages during which social factors are especially consequential for health (43). Along these lines, it is important to investigate whether exposure to systemic racism during certain life stages is particularly harmful for contemporaneous and/or subsequent health outcomes (e.g., scarring effects). Relatedly, future research is needed to assess the extent to which certain forms of systemic racism are more or less salient during particular life stages, which will provide important insights into the relative utility of using age-specific versus general measures of systemic racism. Moreover, we must understand better how the sequences and durations of exposures to systemic racism across the life course affect health outcomes. Finally, life course perspectives and research designs on systemic racism are critical for testing conditions under which individuals experience latency periods, (e.g., time gaps between exposure to systemic racism and manifestations of their health consequences).

### 3.3. Sociohistorical and Geographic Locations

The life course principle of historical time and place can be a valuable tool for studying the effects of systemic racism, given its emphasis on how individuals’ lives are influenced by the eras and locations in which they live (29, 45). Indeed, an important insight from life course research is that individuals born around the same time experience unique sets of conditions that affect their lives in ways that differ from those born during different times (i.e., cohort effects). Thus, we need to examine how specific birth cohorts experience distinct aspects of systemic racism that are likely to impact health (60). For example, individuals born between 1900 and 1940 faced Jim Crow legalized discrimination; those born between 1940 and 1960 grew up during the Civil Rights Era, and those born between 1970 and 1990 came of age during the era of policies that fueled the racialized mass incarceration crisis; all of these experiences may have unique health consequences (81, 126). From a life course perspective, it is also important to study potential period effects to gain a better understanding of how specific racialized policies, events, norms, and circumstances at a point in time shape the health of broad swaths of the population, regardless of stage in life or cohort. Furthermore, when studying phenomena related to race, considering the role of place is critical. By examining different geographical contexts, researchers can compare how systemic racism manifests differently in various places and the implications that this variation has for population health. In sum, the principle of historical time and place is useful for studying the health consequences of distinct sociohistorical contexts of systemic racism.

### 3.4. Linked Lives

The life course principle of linked lives underscores the interconnected nature of the human experience. Individuals’ lives are profoundly impacted by those around them, and social networks are critical contexts through which social conditions affect health (29, 61). Thus, a more complete understanding of the impact of systemic racism on health requires careful analyses of the various relationships through which the harmful effects of systemic racism may be transmitted, including romantic relationships, intergenerational relationships, coworker relationships, and community relationships. For example, studies have begun to document the health-harming spillover effects of racialized incarceration and police violence on women and children (25, 85, 117), even when the primary targets of these forms of systemic racism are typically Black men.

Application of the linked lives principle to the study of systemic racism and health raises many important research questions, such as How do parents’ and grandparents’ systemic racism exposures impact children’s health and life chances? How do systemic racism exposures of friends, family, and community members shape one’s own health and well-being? Do some specific domains of systemic racism have larger spillover effects? The principle of linked lives also informs our theorizing about and interpretations of racism’s health consequences for Whites. For example, to the extent that White lives and Black lives are linked within a given context, we might expect a pattern of universal harm whereby Whites’ health is also undermined by systemic racism because of its damaging effects on social relationships and communal resources and investment (21, 89, 92, 127). Conversely, to the extent that White individuals and communities can unlink their fates from those of their Black counterparts through segregation and hoarding of resources, opportunities, and power, we would expect to observe either a null effect or a beneficial effect of systemic racism on the health of Whites. In sum, consideration of the various ways that lives are linked will provide a richer understanding of systemic racism and its health consequences.

### 3.5. Human Agency

The life course principle of agency emphasizes that individuals are active participants in their own lives, capable of making choices and taking actions that shape both personal and societal trajectories while also being influenced by the social context in which they live (43, 61). This principle underscores several important ideas that have implications for metrics of systemic racism. First, the principle of agency can help demystify aspects of systemic racism that often seem amorphous, abstract, and impersonal by highlighting the choices and actions of specific decision-makers and architects of racialized societies. To advance the understanding of systemic racism, public health research should prioritize the study of individuals and groups actively shaping its manifestations (22, 82). These include policy makers, criminal-legal officials, bureaucrats, employers, social workers, health care providers, and organizations engaged in both perpetuating and countering racism. Second, while systemic racism imposes constraints on individuals’ freedom, individuals are often still able to exert control over their lives to varying degrees, which is evident in their adaptability and resilience in the face of racialized adversity. Indeed, there are numerous examples of individuals resisting forces of racism in an effort to determine the course of their own lives. Third, agentic resistance on an aggregate scale is evident and measurable in the form of collective actions—for example, political movements, racial uprisings, community organizing, coalition building, and antiracist organizations—characterized by “making good trouble” in the struggle against racism.

## 4. ADDITIONAL CONSIDERATIONS FOR MEASURING SYSTEMIC RACISM

As public health research on systemic racism advances, addressing several critical methodological challenges is essential to deepen understanding and enhance the rigor of future studies. Key priorities include the careful selection of appropriate units and levels of analysis, deliberate consideration of statistical controls, expansion of research on understudied populations, and the integration of perspectives from marginalized racial groups through qualitative and mixed-methods approaches. Furthermore, ensuring sufficient sample size, robust study design, and adequate statistical power is crucial for generating reliable and generalizable findings. Given the inherent complexity of systemic racism, it is important to acknowledge that no single measure can fully capture its multifaceted nature. Consequently, triangulating diverse data sources, measurement strategies, and modeling techniques offers a more comprehensive and accurate depiction of systemic racism and its health impacts. Such a multifaceted approach enables nuanced exploration of how systemic racism operates across varying contexts and populations, ultimately guiding the development of more targeted and effective interventions. Below, we elaborate on these critical issues.

### 4.1. Units and Levels of Analysis

Measures of systemic racism have been developed across a range of geographic scales, including census tracts, neighborhoods, commuting zones, public use microdata areas, counties, states, regions, and countries. As scholars have emphasized, the selection of a geographic unit should be guided by the specific research question and the hypothesized mechanisms through which systemic racism operates (68, 71). However, most studies to date focus on a single geographic scale, limiting the ability to capture the multifaceted and hierarchical nature of systemic racism. A critical direction for future research is the deliberate integration of measures of racism across multiple geographic levels, enabling a more nuanced understanding of how these levels function independently and interactively to shape population health outcomes.

### 4.2. Choice of Statistical Controls

While isolating the effects of systemic racism through the judicious application of statistical controls is crucial, it is equally important for scholars to avoid overcontrolling, which can lead to significant underestimation of the health consequences of systemic racism. Given that systemic racism operates upstream of interpersonal and individual-level factors, researchers must carefully distinguish true confounders from variables that function as mechanisms along the causal pathway (73). For instance, if systemic racism adversely affects the health of Black individuals in the United States by constraining access to higher education, controlling for individual-level educational attainment would obscure the relationship between systemic racism and health by inadvertently adjusting for a key pathway through which racism exerts its effects.

### 4.3. Expanding Research on Understudied Populations

Many of the theoretical and empirical foundations for our understanding of systemic racism are drawn from the oppression and marginalization of Black populations in the United States (71). To be sure, the history of racial and ethnic domination and control extends to other racial and ethnic groups, including, but not limited to, American Indian/Alaska Native, Arab, Asian, Native Hawaiian/Pacific Islander, and Hispanic/Latinx populations. Many scholars in the humanities and social sciences have described specific manifestations of racial violence on and control of these groups (12, 36, 49, 64, 83, 113). For example, Seguin & Rigby (115) identified heterogeneity in lynching practices across the United States, not limited to Black populations in the former slave states, but also evidenced among Mexican nationals along the Texas–Mexico border and among Chinese and Japanese populations in the West. Scholars should draw on relevant insights to develop a more robust understanding of the nature of systemic racism across racial and ethnic groups. Such work is urgently needed to expand our analyses of how White supremacy shapes the health of various minoritized racial groups in the United States as well as in other global contexts.

### 4.4. Including Perspectives of Marginalized Racial Groups

Whereas conventional research on links between race and health has often sanitized racial violence and involved superficial, extractive, and opportunistic approaches that led to inaccurate (and potentially harmful) studies, equitable collaborations with community members result in more rigorous research by incorporating the unique experiences and insights of marginalized racial groups (86). Critical race theory underscores the indispensable role of the lived experiences and narratives of marginalized groups in understanding systemic racism (35, 106). Qualitative and mixed-methods approaches to studying the health consequences of systemic racism are well-suited for enhancing our understanding of the processes, contexts, and meanings experienced by individuals (2, 53, 68, 119). These narratives expose the subtle and overt manifestations of discrimination, offering a rich source of data that can be used to identify and quantify systemic inequalities (28). This approach not only enhances the authenticity and comprehensiveness of the measures but also ensures that they reflect the real-world complexities of racism, thereby facilitating the development of effective and targeted interventions (10, 68).

### 4.5. Sample Size and Statistical Power

Most large health surveys contain samples that are 7–10 times larger for White respondents than for Black respondents. These sample size disparities are even larger for other racial and ethnic groups relative to White populations. This sample design/composition privileges the experiences of White respondents and masks the experiences of other groups because with such large samples of Whites there is much greater statistical power to detect even small effects (18, 73). This type of unbalanced sample results in apples-to-oranges comparisons across racial/ethnic groups. Thus, we suggest three possibilities for researchers to attend to these issues: (*a*) Future studies can be designed from the outset with balanced samples containing roughly equal numbers of individuals from the racial and ethnic groups being studied (125); (*b*) existing studies with unbalanced samples can be transformed into balanced samples post hoc using randomly selected subsets of data to ensure similar sample sizes across groups; or (*c*) researchers can use more stringent statistical significance thresholds for the larger groups in unbalanced samples so that the effects identified within the various groups are roughly similar in size (73). In sum, attending to these additional considerations will help ensure a rigorous science of systemic racism and health moving forward.

## 5. CONCLUSION: TOWARD THEORY-DRIVEN MEASUREMENT OF SYSTEMIC RACISM

Public health research holds immense potential to deepen our understanding of how systemic racism creates, perpetuates, and exacerbates racial health inequities, while also informing the interventions necessary to dismantle these inequities. However, this potential risks being undermined if the field continues to produce atheoretical and underconceptualized studies. Such research dilutes the knowledge base, oversimplifies complex theoretical constructs, marginalizes the contributions of race scholars—especially scholars of color—and limits our understanding of the racialized social processes that systematically harm Black and Brown communities (124). As scholars, we face a critical choice: to commit to rigorous, meaningful research that transcends disciplinary boundaries and requires sustained intellectual engagement, or to prioritize the rapid publication of findings that, despite statistical significance, contribute little to advancing understanding. The research we produce not only reflects our values but also shapes the field and its impact. Furthermore, in our roles as reviewers, editors, colleagues, collaborators, teachers, and mentors, we bear a collective responsibility to hold ourselves and each other accountable. Are we meeting this responsibility? Are we doing the necessary work to drive meaningful change?

## Figures and Tables

**Table 1:** Systemic racism features and corresponding measures and research questions

Features of systemic racism	Illustrative measures and modeling approaches	Research questions
Relational hierarchies and power dynamics	(*a*) Power structure analysis of specific racialized actors (e.g., individuals, organizations, institutions, and government entities) using organizational data, surveys, experiments, and network and content analysis to capture who holds power and how it is exercised and perceived (*b*) Racial stratification in power and life chances (e.g., autonomy, political representation, voting rights, homeownership, incarceration), as indicated by administrative, survey, and digital trace data (*c*) Discriminatory policies underlying racial hierarchies (*d*) Racialized ideologies related to beliefs about hierarchies of human value and deservingness	Who are the racialized actors (e.g., ordinary citizens, elected officials, police, corporations, states) that play an outsized role in contributing to racial health inequities? What are the health consequences of how White supremacist power is enacted and perceived? Which discriminatory policies (and bundles of policies) are especially deleterious in terms of health, and how do these effects vary by race? How do racial hierarchies—as measured by racialized ideologies and inequities in power and material conditions—influence population health? To what extent are the effects of relational hierarchies and power dynamics specific to health condition?
Historical roots and amnesia	(*a*) Sociohistorical variation in prevalence of racial violence (e.g., enslavement, lynching, poll taxes, sundown towns, anti-miscegenation, and other forms) (*b*) Racialized regimes characterized by distinct racial structures based on packages of racist phenomena (*c*) Home Owners’ Loan Corporation (HOLC) maps	How do historical forms of racial violence link to contemporary health inequities? How do these relationships vary over time? What intervening factors mediate or moderate these relationships?
Racialized logics and ideologies	(*a*) Survey measures of racial antipathy, resentment, and apathy; polls and voting patterns related to support for racist public officials and racialized policies (*b*) Digital trace data on racism on search engines, social media, and other websites (*c*) Experimental approaches to measuring unconscious and implicit associations and ideologies	To what extent do racialized logics of specific individuals, organizations, and institutions shape population health among racialized minorities? How are the various aspects of racialized logics and ideologies related, and how do they collectively affect health? Under what circumstances do racist ideologies benefit, harm, or not affect the health of White people?
Institutional manifestations	(*a*) Black–White inequality ratio measures in various institutional and organizational domains such as the labor market, criminal justice system, housing, etc. give a broad picture of the many ways racism is embedded in institutions (*b*) Specific laws, policies, and practices such as affirmative action, redlining, wage theft, and immigration policies provide greater detail on how racism actually operates to harm health	How can we best capture the way racism operates in specific institutions? How are logics and practices of racism operating in different institutions similar or different from one another? Is racism in some institutions more or less impactful than in others in terms of health harms?
Ubiquitous and multifaceted nature	(*a*) Including multiple, separate indicators of systemic racism allows for testing of independent, additive, and synergistic effects (*b*) Summative indices measure multiple indicators of systemic racism and assume equal weighting of indicators (*c*) Latent variable scales capture unobserved phenomena, minimize measurement error, and allow for differential weighting of observed indicators	Relative to single indicators of systemic racism, does including multiple aspects of systemic racism in health research enhance predictive capacity? Do various components of systemic racism jointly shape health in an additive or synergistic fashion? Do latent measures of systemic racism account for more variation in health than do single and summative indicators of systemic racism? If so, are there conditions under which this is not the case?
Interconnected and adaptive network	(*a*) Agent-based modeling simulates interactions of autonomous agents (e.g., individuals, organizations, and institutions) to assess systems and their emergent properties (*b*) System dynamics modeling analyzes feedback loops and time delays to understand the behavior of complex systems over time (*c*) Machine learning methods are effective for analyzing large datasets and complex patterns	What are the key links among systemic racism components for understanding health inequities? To what extent do components of systemic racism have reciprocal, feedback, and lagged effects? How does systemic racism adapt over time, and what are the health implications?

**Table 2:** Time matters: life course approaches to studying how health is shaped by systemic racism

Life course principle	Illustrative approaches	Research questions
Lifelong human development	(*a*) Measure systemic racism longitudinally (*b*) Operationalize transitions (discrete changes) and trajectories (long-term patterns of stability and change) in systemic racism exposure (*c*) Model systemic racism pathways (e.g., chains of risk) to study sequences of linked systemic racism exposures	How does cumulative exposure to systemic racism across the life course affect developmental processes? To what extent does exposure to systemic racism earlier in life lead to a cascade of risks, resulting in an accumulation of social, economic, and health disadvantages? How is later-life health shaped by exposure to systemic racism in early, middle, and later life?
Timing in lives	(*a*) Test for sensitive periods (*b*) Estimate models that include both early-life and later-life racism exposures when possible to examine the unique and combined health effects (*c*) Utilize age-specific systemic racism measures (*d*) Explore latency periods between exposure and outcomes by testing time lags	Is systemic racism exposure during certain life stages (e.g., in utero, childhood, adolescence, early adulthood) especially deleterious? Which aspects of systemic racism have immediate, persistent, and/or lagged effects on health? Is systemic racism in domains that are directly relevant to one’s life stage (e.g., for children, racism in schools) particularly consequential for health? Are the health effects of prior racism exposures buffered by subsequent exposures to more racially equitable environments, or is there evidence of scarring effects?
Sociohistorical and geographic locations	(*a*) Operationalize cohort effects to study how individuals born around the same time experience unique sets of racialized conditions (*b*) Examine period effects to understand how specific racialized phenomena at a point in time influence the broader population, regardless of age or cohort (*c*) Measure place-based aspects of racism (i.e., different racial regimes)	How does cumulative exposure to systemic racism—and its relationship with health—vary across birth cohorts? To what extent do specific racialized policies (e.g., voting rights and anti–critical race theory laws), social movements (e.g., Civil Rights, Black Lives Matter, Make America Great Again), events (e.g., high-profile killings of unarmed people of color), and norms (e.g., prevailing racial ideologies and practices) at a point in time affect health similarly for various age groups and cohorts? How do racial regimes vary across geographic locations, and what are the implications of this place-based variability for health trajectories?
Linked lives	(*a*) Investigate how the effects of systemic racism are transmitted through social relationships (*b*) Examine romantic relationships, intergenerational relationships, coworker relationships, and community relationships (*c*) Employ social network analysis as well as qualitative data collection	How do systemic racism exposures of parents and grandparents impact children’s health and life chances? How do systemic racism exposures of friends, family, and community members shape one’s own health? To what extent are health effects of vicarious racism contingent upon racial identities and linked fate? Which aspects of systemic racism have the largest spillover effects on health?
Human agency	(*a*) Measure race-related forms of resilience and resistance (*b*) Examine forms and consequences of collective action (*c*) Employ racialized power structure analysis of agency among individuals and organizations (*d*) Examine White normativity in actions and inactions	How does systemic racism exposure affect resilience? What types of personal and social resources can help buffer the harmful health effects of racism? How do different forms of antiracist collective action impact individual and community health? What forms of collective action are most efficacious for advancing racial health equity? Which racialized actors are best positioned to exacerbate or ameliorate the deleterious effects of systemic racism?

## WHAT IS SYSTEMIC RACISM?

Systemic racism is a complex, variegated, dynamic phenomenon that defies a simplistic definition. That said, in general, systemic racism refers to the unequal allocation of economic, social, political, legal, cultural, psychological, and health rewards along racial lines in a society (10, 16, 47, 95, 106). The analytical crux for understanding systemic racism is identifying the collective logics, properties, practices, and mechanics that create and perpetuate racial domination (13, 21, 48, 57, 58, 85, 95, 103). These elements constitute the racialized structure of society and manifest in specific racial regimes, such as chattel slavery, Jim Crow segregation, and contemporary forms of new racism (13). However, simply incorporating a definition of systemic racism into health studies is insufficient without a clear understanding of its theoretical and analytical implications. Researchers must critically interrogate the conditions under which racism exists, the agents and institutions that enact it, the beneficiaries of its outcomes, the mechanisms through which it operates, and the processes that ensure its reproduction over time. To address these complexities, we draw on interdisciplinary theory and evidence to dissect the core features of systemic racism—such as relational power dynamics, historical roots and amnesia, racialized logics and ideologies, institutional manifestations, its ubiquitous and multifaceted nature, and its interconnected and adaptive networks—and discuss their implications for operationalization in research. It is also important to acknowledge that terms such as “systemic,” “structural,” and “institutional” racism are often used interchangeably. While some scholars treat these terms as synonymous, others differentiate among them to highlight distinct dimensions and processes of racial inequity (13, 103). For the purposes of this article, we conceptualize systemic racism as an overarching phenomenon that encompasses multiple forms of racism, including but not limited to structural, institutional, interpersonal, cultural, and internalized racism. It operates both within and across micro, meso, and macro levels, shaping and reinforcing racial inequities through interconnected and multilevel mechanisms.

## SUMMARY POINTS

1. Public health research on the health consequences of systemic racism must consider the structure and function of systemic racism: why racism exists, who enacts it, who benefits from it, how it works, and how it is reproduced.
2. Approaches to measuring and modeling systemic racism should align with interdisciplinary theories of racism.
3. Theories highlight core features of systemic racism that are relevant for measurement: relational hierarchies and power dynamics; historical roots and amnesia; racialized schemas, logics, and ideologies; institutional manifestations; ubiquitous and multifaceted expressions; and interconnected and adaptive networks.
4. Incorporating life course principles will advance the study of systemic racism and its health consequences by centering dynamic, temporal processes.
5. Key life course principles are lifelong human development, timing in lives, historical time and place, linked lives, and human agency.
6. The life course perspective points to the intricate interplay of racialized biographical, historical, and societal trajectories, emphasizing various dimensions of temporality such as age, period, cohort, and intergenerational factors.
7. Researchers should carefully consider units and levels of analysis; choice of statistical controls; expansion of research on understudied populations; inclusion of perspectives of marginalized groups; and sample size, design, and statistical power issues.
8. While no single operationalization fully captures systemic racism, triangulating diverse data sources, measurement techniques, and modeling approaches offers a more accurate and comprehensive understanding of systemic racism’s complexities and the effects it has on health outcomes.

## FUTURE DIRECTIONS

1. Modeling dynamic, reciprocal relationships between ideological and institutional aspects of racism is crucial for identifying the intricate mechanics that sustain racial inequalities.
2. Scholars should leverage novel data sources and advanced analytical tools such as agent-based modeling, system dynamics modeling, and machine learning to gain a more comprehensive understanding of complex relationships among various components of systemic racism and their health effects.
3. Measuring distinct racialized regimes across sociohistorical and geographic contexts is essential for comprehending the health effects of varied manifestations of racism and for developing targeted interventions, including in the context of public health, to address racial inequities in diverse settings.
4. Estimating the extent of racialized institutional isomorphism and its consequences for health equity can provide valuable insights into how uniformity in discriminatory practices across institutions affects disparities in health outcomes.
5. Testing the utility of age-specific measures of systemic racism is necessary to understand how systemic racism differentially impacts individuals at various life stages, thereby informing more effective and age-appropriate policy and intervention strategies.
6. Most studies on systemic racism are based on the experiences of Black populations. It is essential to develop novel measures of systemic racism that capture the subordination and exclusion experiences of other racial groups.
7. We recommend that the Department of Health and Human Services support the building and maintenance of a comprehensive, user-friendly data infrastructure on systemic racism. Centralizing high-quality, novel systemic racism data spanning societal sectors and spatiotemporal contexts would catalyze much-needed research on the relationship between systemic racism and health (20).
8. Researchers should examine how systemic racism intersects with other systems of oppression, such as sexism, nativism, ableism, economic exploitation, and LGBTQ discrimination, to better understand and address multifaceted societal forces shaping health.
